# A qualitative exploration of participant and investigator perspectives from the TRED‐HF trial

**DOI:** 10.1002/ehf2.13524

**Published:** 2021-08-13

**Authors:** Vasiliki Papageorgiou, Kathryn Jones, Brian P. Halliday, Richard Mindham, Jane Bruton, Rebecca Wassall, John G.F. Cleland, Sanjay K. Prasad, Helen Ward

**Affiliations:** ^1^ Patient Experience Research Centre, School of Public Health Imperial College London London UK; ^2^ National Heart and Lung Institute Imperial College London London UK; ^3^ Cardiovascular Research Centre and Cardiovascular Magnetic Resonance Unit Royal Brompton Hospital London UK; ^4^ Royal Brompton and Harefield NHS Foundation Trust London UK; ^5^ Robertson Centre for Biostatistics University of Glasgow Glasgow UK

**Keywords:** Cardiomyopathy, Dilated, Feasibility studies, Medication adherence, Patient participation, Physician–patient relations, Qualitative research

## Abstract

**Aims:**

We explored the experiences and motivations of participants and staff who took part in the TRED‐HF trial (Therapy withdrawal in REcovered Dilated cardiomyopathy).

**Methods and results:**

We conducted a qualitative study, using semi‐structured interviews, with participants (*n* = 12) and the research team (*n* = 4) from the TRED‐HF trial. Interviews were carried out in 2019 and were audio‐recorded and transcribed. Data were managed using NVivo and analysed using framework analysis. A patient representative provided guidance on the interpretation of findings and presentation of themes to ensure these remained meaningful, and an accurate representation, to those living with dilated cardiomyopathy. Three key themes emerged from the data: (i) perception of health; (ii) experiences and relationships with healthcare services and researchers; and (iii) perception of risk. Study participants held differing perceptions of their health; some did not consider themselves to have a heart condition or disagreed with the medical term ‘heart failure’. Relationships between participants, research staff, and clinical management teams influenced participants' experiences and decision making during the trial, including following clinical advice. There were differences in participants' perceptions of risk and their decisions to take heart failure medication after the trial was completed. Although the original TRED‐HF trial did not provide the results many had hoped for, a strong motivator for taking part was the opportunity to withdraw medication in a safely monitored environment which had been previously considered by some participants before. Investigators acknowledged that the insights gained from the study can now be used to support evidence‐based conversations with patients.

**Conclusions:**

For people whose dilated cardiomyopathy is in remission, decisions to continue, reduce, or stop their medication are influenced by perceptions of personal health, perceive risk and the important of work, employment, recreation, relationships, and long‐term plans. The unique relationship between patient and cardiologist provides opportunities to promote honest discussion about adherence to medication and personalized long‐term management.

## Patient's perspective

By Richard Mindham

For the relatively young patient, having a chronic disease defines your sense of self and your very being. Taking tablets several times a day, visiting the General Practitioner (GP) for routine tests and repeat prescriptions, and collecting medicines, all act as repeat reminders of being ‘other’. Should providence shine on you and your condition improve, it is common to wonder whether it is possible to shed the sense of being a lifelong patient by reducing or removing your medication altogether. This question was addressed in the TRED‐HF trial where a small number of ‘recovered’ dilated cardiomyopathy patients withdrew their medication under supervision. This paper looks at their experiences and the psychological consequences of success or the unwanted confirmation that they needed to return to their drugs. Their perception of the risk of withdrawal, before and after the trial, is also reported.

## Introduction

Pharmacological therapy for dilated cardiomyopathy (DCM) may lead to resolution of symptoms and normalization of cardiac function and heart failure biomarkers.^1^ Such patients often ask health professionals whether they can stop taking medication.^2^ The ‘Therapy withdrawal in Recovered Dilated cardiomyopathy—Heart Failure’ (TRED‐HF) trial was undertaken to address this question.^2^ The trial found that 40% of participants relapsed within 6 months of beginning withdrawal of therapy, suggesting that their disease had been in remission rather than cured.^2^ The study team, and others,^3^ therefore, recommended that all participants should resume medication until reliable predictors of relapse could be identified.

Whether patients decide to take medications is influenced by many factors, including how they perceive the diagnosis and risk associated with it. A diagnosis of ‘heart failure’ has been shown to lead to denial of illness as a coping mechanism.^4^, ^5^ Other important factors include perception of over‐medicalization and the nature of the relationship with their physician.^6^, ^7^, ^8^ Patient's trust in healthcare providers results in greater treatment adherence,^9^ but whilst collaborative relationships enhance care and sustained contact improves outcomes,^10^ there is a risk that patients perceive that healthcare staff are in control and so fail to make decisions for themselves.^11^ People with DCM are often young and are frustrated by the thought of taking medication for decades with side effects, perceived or actual, which can affect their sense of wellbeing and finances.^2^ Patients become uncertain about whether medications are still required when they become asymptomatic but may also worry about the risk of relapse.

We explored the reasons why patients agreed to participate in the TRED‐HF trial^2^ and their perspectives of the experience. We also sought the views of researchers involved at various stages of the trial including conceptualization and implementation.

## Methods

### Study design

We conducted a qualitative study of participants and researchers involved in the TRED‐HF trial using framework analysis.^12^ The study was informed by phenomenology, in particular, describing and understanding the meanings of lived experience.^13^, ^14^ We provide further detail of our method in *Supporting information* *Data S1* and report our findings using the standards for reporting qualitative research (SRQR) checklist (*Data S2*).^15^

### Sampling

The TRED‐HF lead researcher (B. H.) categorized participants based on study outcome into three categories: (i) relapsed; (ii) did not relapse, followed recommendations to go back on medication; (iii) did not relapse, did not follow the recommendation to go back on medication. Two researchers (V. P. and K. J.) randomly selected 15 participants (five from each category) using a computer‐generated number, reviewed the selection for a mix of age and sex in each category and reselected as required to reflect the study population. Two participants who did not fit the categories were also included. B. H. selected six members of the research team to participate. All potential participants were sent an information sheet and provided informed consent for the sub‐study.

### Data collection

Two researchers (V. P. and K. J.) interviewed 12 TRED‐HF study participants and four study staff between March and July 2019 using a topic guide informed by available literature (*Data S3*). Participants were asked about their motivations for taking part, experience during the study, and life since it had ended. Staff were asked about their role during the study, interactions with participants, and reflections. Interviews were audio‐recorded and transcribed verbatim by a professional service.

### Data analysis

Transcripts were uploaded and managed using QSR International's NVivo 12 software and analysed by V. P. and K. J. using framework analysis.^12^, ^16^ Transcripts were coded, indexed, and charted within a framework of timepoints from trial participation; before, during, and after. A short description for each participant was noted under each theme, to capture the meaning or context.^12^, ^13^ The framework matrix was discussed between researchers (V. P., K. J., H. W., and J. B.) and using a thematic approach, commonalities and exceptions were identified.^13^ These themes were finally contextualized and interpreted using the current literature and a reflexive approach.

### Patient and public involvement

Patients were involved throughout the conceptualization, design and conduct of the original TRED‐HF trial. We presented the initial findings of this study to a Patient Advisory Group in June 2019 and worked with RM for advice on interpretation and refinement of themes from the data, based on his lived experience.

### Ethical approval

The study complies with the *Declaration of Helsinki*. Ethical approval for this study was obtained, as a sub‐study (IRAS ID: 171308) of the original TRED‐HF trial, from the National Research Ethics Committee (16/LO/0065). Informed consent was received from all participants.

## Results

The demographics of participants were similar to those in the original TRED‐HF trial.^2^ Seven were men and five women, 11 were White British and one Black British/African. Median age on enrolment to TRED‐HF was 46 years (*43–62 interquartile range 19*) and at interview 49 years (*46–64 interquartile range 18*). Four participants were categorized as ‘relapsed’, three ‘not relapsed, back on medication’, four ‘not relapsed, not back on medication’, and one had withdrawn from the study. Eleven interviews took place in person and five by telephone. Study staff interviewed were the principal investigator, co‐ principal investigator, research fellow, and a doctor analysing blinded cardiovascular magnetic resonance images.

**Figure 1:**
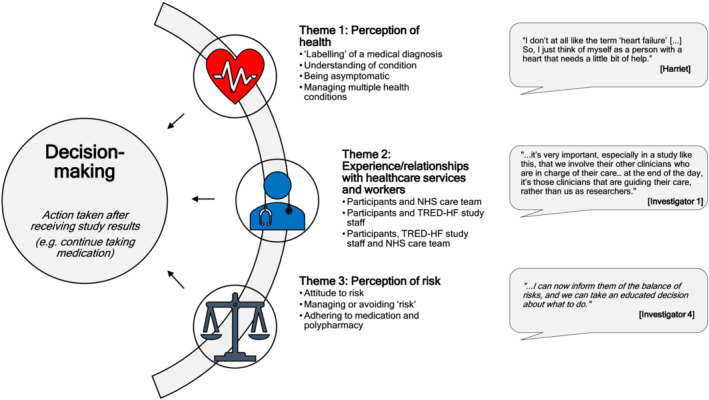
Summary of main themes and sub‐themes from the analysis of interview transcripts. Pseudonyms are given for participants to maintain anonymity.

We present data for three major themes and sub‐themes (*Figure* *1*) and have used pseudonyms for participants interviewed. Our framework analysis (*Data S4*) presents the themes, which summarize the findings from interviews with all participants, within the context of the ‘illness and treatment’ and TRED‐HF study pathway.

### Motivation for participation

Motivations to take part in the TRED‐HF trial focused on personal gain, such as stopping or reducing pill ‘burden’, and altruism, to help others with the condition (including affected family members and future patients). Participants with fewer time commitments, for example, those who were retired, described a sense of duty to take part to advance knowledge and assist the health service. In contrast, a younger participant described the process as *time‐consuming* and *disruptive* for their work.

### Theme 1: Perceptions of health

We asked participants to tell us about their heart condition, which led to a discussion on their diagnosis, what this means to them, the asymptomatic nature of their current DCM, and managing this in relation to other health conditions.

#### ‘Labelling’ of a medical diagnosis

Most explicitly described themselves as having a heart condition; however, one felt her heart had since ‘recovered’. Some did not view themselves as having a heart condition; their heart had ‘healed’ or was secondary to a previous self‐limiting infection. Participants referred to ‘labelling’ of medical terminology. For some, receiving a diagnosis harmed their wellbeing; Ella described initially feeling *psychologically crushed* with her *‘*… hopes and dreams of living a really long life were very questionable… It can make you quite depressed, although you keep it to yourself most of the time.’

#### Understanding of condition

Participants thought trial visits were helpful to improve their understanding of their condition and receive more attention than would occur during routine care. Feeling comfortable with the level of attention and interacting with a ‘new’ clinical team differed; Alan described feeling embarrassed about receiving greater attention during the trial when he otherwise felt well, whereas Jack was concerned that researchers might be less familiar with his condition than his regular clinical care team.

#### Being asymptomatic

Some participants appeared to have a disconnect between their perceived and actual health, likely linked to the largely asymptomatic nature of their condition. For instance, Daniel explained that the condition does not affect his day‐to‐day life, although he does sometimes experience short‐lived chest pain, which is thought to be non‐cardiac in origin. After withdrawing from the trial shortly after enrolment, and despite always remaining on medication, he was told that his heart function had deteriorated slightly, and he was surprised by this. Some described that others do not see any visible signs of illness, which impacts how they perceive their health. Some were uncertain of the cause of their condition, which made them worry about the potential for symptoms to return once medications were stopped.

#### Managing multiple health conditions

Participants living with multiple health conditions described a sense of frustration when navigating the healthcare system and managing medications. For example, Katie described negative experiences of talking about managing her diabetes with cardiologists she had previously encountered often working in ‘silos’. She wanted a more open and honest, rather than dismissive, conversation with health professionals. She felt that her relationship with study staff was more positive, ‘[Name] didn't do that; that's just the general cardiology world that I've interacted with.’

### Theme 2: Experiences and relationships with healthcare services and workers

We explored relationships between participants, study staff, and the clinical care teams, which are summarized in *Table* *1*.

**Table 1:** Quotes from interview transcripts highlighting relationships before, during, and after the TRED‐HF trial

Trial timeline	Context	Quote	Codes	Themes
Before	Ella discusses her initial delays with being diagnosed by her clinical care team.	‘So, they've missed me for years, down here. There's obviously something going on, and they wouldn't listen to me.’ [Ella]	‘Dismissive’ healthcare staff; delayed diagnosis	Participants and clinical care team
During	Katie describes her relationship with TRED‐HF investigators during the monitoring stage of the study	‘So, I was emailing [Name] late at night, he was calling me back even later and giving me some information about my previous scans that I'd had, which actually helped with what was going on.’	Open communication; feeling involved; updated health status	Participants and TRED‐HF investigators
	Iris described tense interactions with receptionists at her primary care practice whom she felt were not always aware of the changes to her dosages, despite regular communication from the research team and her usual clinical team.	‘It was very procedural for them (primary care receptionists), and this didn't fit into their procedure. They weren't able to be flexible. You know, “Oh, you've just changed. You changed your dose three weeks ago. I'm not putting this in front of the doctor again,” and I'm like, “Oh, please, because I'm trying to chop them up, but I can't…”’	Managing dosage; navigating healthcare system; negotiating care	Participants, TRED‐HF investigators, and clinical care team
	Investigator 1 emphasizes the importance of liaising with participants' clinicians during the trial.	‘… it's very important, especially in a study like this, that we involve their other clinicians who are in charge of their care… at the end of the day, it's those clinicians that are guiding their care, rather than us as researchers.’	Collaboration; patient‐provider relationships; shared decision making	Participants, TRED‐HF investigators, and clinical care team
After	Iris outlines her feelings after the TRED‐HF trial had ended. She was under the care of her usual clinical care team and had been recommended to restart medication by the research team.	‘… I'm a little bit worried because I feel all at sea. I feel a bit off‐piste now, and I'm starting to feel a bit more symptomatic, but I don't know if it's psychosomatic, which I don't. I just feel a bit… I don't know what to do now because I feel like I'm not on their books anymore.’ ‘What's almost made it worse that I've been on the study is you've had that glimpse. You've kind of seen the other side, and then you've got to go back on it…’ [Iris]	Sense of abandonment; uncertainty of treatment plan; managing symptoms	Participants, TRED‐HF investigators, and clinical care team
	Christine describes a tension with her clinical team when deciding whether to follow the research investigators' advice to go back on medication.	‘At the moment I'm stuck between a rock and a hard place, between one hospital saying, “I'm not really sure,” and one hospital advising that maybe it's a good idea to go back on and the GP convinced I should go back on…’	Navigating healthcare; patient‐provider relationship	Participants, TRED‐HF investigators, and clinical care team
	Investigator 3 highlights the importance of a patient‐centred approach to making decisions about care.	‘If they've got capacity, it's their choice. I always feel as a doctor, my job is to explain or let people know what the current evidence base is, what the recommendation of the physician would be… for them to take that information and think about how it fits in with their life and then make the choice that they want to make’	Shared decision making; patient‐centred care	Participants, TRED‐HF investigators, and clinical care team

### Theme 3: Perceptions of risk

#### Attitude to risk

Participants' perception of, and attitude to, risk appears to have influenced their decision to take part in the study as they weighed up whether their health would be better or worse following the trial. However, they also described the risk of potential consequences for, or opinions of, loved ones. Younger participants, or those with dependents, described balancing risks to their health in taking part, with the hope of not having to take lifelong medication, and the impact on the lives of their families.

#### Managing or avoiding ‘risk’

Participants described the study as being a ‘safer’ way to stop or reduce the amount of medication as they would be closely monitored. Monitoring was thought to ‘minimize’ or ‘avoid’ any potential risks by those who felt daunted or apprehensive about taking part.

Harriet explained that she had thought about stopping medication herself but that her consultant had explained that the study would be a ‘better way’. Additionally, Katie knew that deciding to take medication was her choice but that she usually followed the advice of medical professionals. This seemed to be centred around her role as a mother and potential risk of being non‐adherent,

… But I could've just stopped the medication and be done with it. But there's too many others […] now in my life. I've got a child; I can't play with my life like that…

Investigator 4 commented on the value of the study in managing risk and how this can help to shape two‐way conversations with patients they support,

…I can now inform them of the balance of risks, and we can take an educated decision about what to do.

#### Adhering to medication and polypharmacy

Adherence was affected by having to manage other chronic conditions, experiencing side effects, feeling fit, and personal choice. Most had a clear understanding of when and why medication was necessary; however, those managing more than one lifelong condition described the worry and disruption of polypharmacy,

I was on medication for the unforeseeable future. I guess, that didn't sit well with me, I don't particularly want to‐ because I'm a very forgetful person anyway… [Jack]

Some specified the ‘burden’ of the amount and duration of medication,

‘I mean I don't have a problem taking meds, [because it's just] in the morning, they're in the pill box and I just take them. But I was taking, like, seven separate tablets a day and I just thought, ‘Do I really want to do this for the rest of my life?’ [Greg]

Some participants had experienced side effects from medication: *muzziness*, lacking energy, feeling *zonked out*, or other non‐specific symptoms [Daniel, Iris, Katie, and Bill].

Side effects may affect medication adherence, or how doses are managed; Daniel referred to a routine check‐up finding that his heart function had deteriorated; he declined his heart specialist's desire to increase dosage ‘because of the side effects’. Jack described intentionally not taking his medication prior to the study whenever he *felt fine*. However, most participants described motivations to take part in TRED‐HF driven by the amount of medication, rather than experiences of side effects.

Others described feeling the medication was ‘unnecessary’, particularly if they felt well, and often linked this to medical staff prescribing tablets that were not needed (or ‘medicalization’). Frank described his primary care physician as “a little prescription happy”, and another did not understand why medication was necessary,

But again, at the same time I'm still a bit dubious about it because if I don't have signs and symptoms and my heart is okay, why do I need to go back on the medication? [Christine]

Finally, Christine described being adherent to medication if, overall, it protected her health, considering ‘taking a pill every morning and every evening’ was not invasive.

### Action taken after receiving study results

We explored whether participants decided to follow the study's recommendation to restart medication. We compared each participant's self‐reported outcome to their initial category during the sampling process to see whether these were consistent (*Table* *2*).

**Table 2:** Outcome categories concerning medication adherence reported before and during interviews

Participant outcome categories before interviews (by study staff)	Self‐reported medication adherence during interviews (by participants)
4 relapsed	4 taking medication (1 same dose; 1 less medication; 1 lower dose; 1 did not report dosage)
3 did not relapse, back on medication	3 taking less medication
4 did not relapse, not back on medication	2 not taking medication, 2 taking medication (1 reporting side effects; 1 taking lower dose but self‐medicating)
1 withdrew early	1 taking medication

Most participants followed the advice given to restart medication. Any differences in reporting could be either participant inaccuracy but more likely a time delay between interviews and their last contact with study staff.

Iris described managing her medication regime and taking responsibility for the risk of doing so,

I haven't properly gone back on it the way I should, and the way that has been recommended, so I take full responsibility for that because I guess I'm kind of waiting until I have my appointment, and I'm hoping they say to me, Actually, it's fine, so you don't have to.

Despite this, she explains her hope of a personalized medication schedule to find ‘the right dose for me’ highlighting the individualized nature of taking part in research. Trial staff commented on the value of the study in developing a rationale and evidence:

As clinicians, we're asked a lot by patients… whether they can stop taking medications… in the past, we've based our answers to patients based on anecdotes, rather than… robust evidence. [Investigator 1]

I'm glad that we've got an evidence base for this question because there was no evidence base. People would ask you in clinic and you could say something like current practices, but current practice is just what people do. [Investigator 3]

Investigator 4 explained how the study answered some questions and raised others but will ultimately enable more transparent and well‐informed conversations,

… I think that it helps us already to have a better discussion with patients about risks and benefits of treatment withdrawal.

When asked what future research should cover; participants suggested research into the causes of cardiomyopathy, managing multiple conditions (e.g. a heart condition together with diabetes or thyroid problems), drug research (e.g. exploring benefits, side effects as well as new medications, and alternative treatments). Similarly, study staff suggested determining the significance of treatments, whether there are acceptable alternatives and investigating the underlying mechanisms or causes of the condition. Staff also suggested closer investigation of patients who did not relapse and those with a genetic predisposition.

## Discussion

This study found that a strong driver for patients agreeing to participate in TRED‐HF was to address an unanswered question, in a safely monitored environment, ‘what will happen if I stop taking my medication?’ Participants took part with the hope of getting the answer they wanted for themselves; the hope of reducing medication (dosage and quantity) was especially evident when participants did not view themselves as having a heart condition or felt their heart had ‘recovered’. This is likely to have been influenced by the study's conceptualization through patient demand and design informed by public involvement.

The TRED‐HF trial did not provide the results many participants hoped for (i.e. that medication could be stopped), but most participants appeared to follow the recommendation to restart medications after the trial. Some had decided to make their own decisions; for example, remaining on a lower dosage, or to continue not to take medication. The decision to continue taking heart medication was driven by several factors including managing other health conditions together with views on subsequent polypharmacy, being asymptomatic and fearful of symptoms returning, and attempting to navigate an often complex and disjointed wider healthcare system. Similar challenges have been described among people treated for latent tuberculosis^17^ and people living with HIV.^18^ Decision making is therefore largely underpinned by an individual's perceptions of risk. However, TRED‐HF participants may be less risk averse and keen to stop taking medication compared with other patients with recovered DCM.

Recovered DCM provides an interesting narrative because most patients do not have symptoms of heart failure, yet it is a lifelong condition. One study^5^ describes a deliberate or unconscious process of denial of illness to cope with a heart failure diagnosis. Patients may, therefore, view DCM in a similar way to patients with other ‘stable’ conditions that are perceived as ‘cured’ (e.g. breast cancer survivorship), after normalization of cardiac function.^19^ This contrasts with the needs of those who require treatment to control the symptoms and signs of heart failure. Lehman *et al*.^4^ have suggested re‐labelling the term ‘heart failure’ as ‘cardiac impairment’ to avoid the patient thinking that ‘failure means the end of hope’, which might have a negative impact on adherence to treatment.^20^ The study population of TRED‐HF living with DCM was younger than the broader population of heart failure patients; we found evidence of psychological implications of a DCM diagnosis and prescribed medications such as view of life and mortality. Taking daily medication was a reminder of living with a chronic condition. The original TRED‐HF trial also included symptom burden assessments using the Kansas City Cardiomyopathy Questionnaire (KCCQ) and Symptom Assessment Questionnaire (SAQ) and found no significant difference between randomized groups.^2^, ^21^

A strength of the study is that we were able to understand the reality faced by people living with a largely asymptomatic, yet lifelong condition. Working in collaboration with clinical researchers, we quickly built a rapport with interviewees to explore complex topics including adherence to medication. We were also able to understand how and why patients may choose not to follow advice after a trial has finished. We felt that because the interviewers (V. P. and K. J.) were not involved in the original TRED‐HF trial, we were able to elicit more honest responses from participants. However, our study remains limited by its context‐specific nature particularly as this was conducted in an National Health Service (NHS) setting and we interviewed a small sample of the original study population. A qualitative study design means our results must be considered as ‘hypothesis generating’. We are also unable to comment on whether theoretical saturation was met (i.e. whether any new data would have emerged from interviewing additional participants), as each clinical trial experience was as unique as the previous; however, we feel that a range of backgrounds and experiences were captured in this study.

TRED‐HF has shifted the narrative around traditional, and often more paternalistic, approaches to medicine to person‐centred care. The results should inform patients' decision to adhere to medication but also to clinical advice. For instance, this could be by providing patients with the space and time to ask honest questions free from judgement whilst being empathetic to the potential implications of the outcomes of clinical investigations to the patient's ‘lifeworld’ as is common practice in some other therapeutic areas.^22^, ^23^ Future studies should consider the potential psychological impact of ‘disappointing’ research findings on their participants. This should be co‐created with all stakeholders (patients, carers and clinical staff) involved across the diagnosis, treatment and care pathway, in a way that acknowledges power differentials and governance structures, to optimize research impact.^24^ Despite the efforts of the TRED‐HF trial team of communicating with primary care physicians, cardiologists and participants, we found that some study participants struggled with the transition back to standard care and accepting disappointing study results.

## Conclusions

Patients with DCM and heart failure who are in remission perceive their health in different ways. This is influenced by previous interactions with healthcare professionals, personal relationships, and their hopes and expectations for the future. Whilst surprising for caregivers, for some patients, the concern regarding side effects and the impact of taking medication on quality of life may be greater than the risk of heart failure relapse. A proportion remains eager to stop or reduce medication and may resort to self‐management against advice, even equipped with the knowledge of the high risk of relapse observed in the TRED‐HF trial. To ensure the best outcome for patients, we must move from a paternalistic, ‘one size fits all approach’ to an open shared decision‐making approach, including candid discussions about the risk of relapse and taking into account the individual's perception of risk and hopes for the future.

## Conflict of interest

R. M. received an involvement payment (in line with NIHR Centre for Engagement and Dissemination guidance), which was subsequently donated, in full, to Cardiomyopathy UK.

## Funding

This work was supported by the British Heart Foundation (grant number FS/15/29/31492); the Alexander Jansons Foundation; Cardiovascular Research Centre and NIHR Biomedical Research Unit at Royal Brompton Hospital and Imperial College; Imperial College Biomedical Research Centre; the Wellcome Trust (grant number 107469/Z/15/Z); and Rosetrees Trust.

## Author contributions

V. P., K. J., B. H., S. K. P., and H. W. contributed to the design of the interview topic guide. B. H. coordinated recruitment to interviews. V. P. and K. J. conducted interviews and analysis of results and drafted the manuscript. H. W., R. M., J. B., S. K. P., and B. H. informed the analysis of results. All authors read and approved the final manuscript.

## Supporting information


**Data S1.** Further detail on qualitative methods.

**Data S2.** Standards for reporting qualitative research (SRQR) checklist.

**Data S3.** Topic guide used with TRED‐HF participants and staff interviewed.

**Data S4.** Diagrammatic representation of main themes and sub‐themes identified in framework analysis.
