# Supplementary material for: A qualitative exploration of participant and investigator perspectives from the TRED‐HF trial
Source: ESC Heart Fail. 2021 Aug 13;8(5):3760–8. doi: 10.1002/ehf2.13524 (PMC8497205; doi:10.1002/ehf2.13524)
Supplement: Supplementary file 1 — Data S1. Further detail on qualitative methods. [file EHF2-8-3760-s004.docx]

### Supplementary File 1: Further detail on qualitative methods

Two researchers (VP, KJ) interviewed 12 study participants and four staff between March and July 2019. The interviews took place in a private room at the study site (n=11: eight participants, three staff), or by phone (n=5: four participants, one staff), using a topic guide **(Supplementary 3)**.

Participants were asked about their motivations for taking part in the study, their experience during the study, and life since the study ended. We were also interested in finding out whether they had since followed the study’s advice to continue to take medication. Staff were asked about their role during the study, interactions with participants and reflections. We spoke with staff involved at all stages of the research including those involved at the initial design stage to those responsible for monitoring the health of patients whilst on the study using scans.

Interviews lasted between 14 and 43 minutes. Audio recordings were transcribed verbatim by a professional company of which Imperial College London has a license agreement (which covers confidentiality) established. Audio recordings held by the research team were destroyed following transcription and all transcripts are password-protected and held on an encrypted server of Imperial College London. We have reported all quotes using the exact phrasing used by participants to report their thoughts and feelings in their own words.

**Data analysis**

Transcripts were uploaded and managed using QSR International’s NVivo 12 software and analysed by VP and KJ using framework analysis.^1,2^ Framework analysis is similar to a thematic analysis by which transcripts are initially coded, labelled (or indexed) and then charted (or synthesised into a chart) beneath identified themes and sub-themes.^2^ In our analysis, we used a framework of the time points from TRED-HF study participation (before, during and after) whereby each row represented a person interviewed and each column a theme. A short description for each participant was noted under each theme, to capture the meaning or context.^2,3^

The framework matrix was discussed between researchers (VP, KJ, HW, JB) and using a thematic approach, commonalities and exceptions were identified.^3^ These themes were finally contextualised and interpreted using the current literature and a reflexive approach; reflexivity is the process by which a researcher (in this case VP, KJ) considers how their role may affect how and why the information was and was not shared by a participant but also how their role may influence how the information shared is interpreted.^2^ For instance, as the interviewers’ were not part of the original TRED-HF study, some participants may have felt able to be more honest and open about their experiences, including any that were negative. This is further described in the section on positionality below.

An example of an initial framework analysis of part of a participant’s interview transcript is highlighted in **Table 3** below.

**Table 3:** An example of a framework analysis of an individual participant's responses.

N.B This is a section of the analysis of a participant’s transcript and any identifiable information, including characteristics, have been omitted.

| **Interview participant** | **Before TRED-HF study** | **During TRED-HF study** | **After TRED-HF study** | | **Cross-cutting** | |
| --- | --- | --- | --- | --- | --- | --- |
| [Participant ID] and characteristics (e.g. outcome group, sex, age, ethnicity) | A: Designing the study and recruitment | B: Monitoring and patient safety | C: Study results and follow-up | D: Future research | E: Perception of health and experience of healthcare services and providers | F: Perception of risk and medication compliance |
|  | **Motivation (less medication)** Describes not wanting to be on *''as many pills for the rest of my life as I was on at the time''. ''It makes sense if somebody has prescribed these drugs to somebody, they need to take them. However, I was perfectly happy in a controlled environment where you then withdraw that medication''.*  **Perception of risk**  *''At what point would I have said no to it? It’s a difficult question. If I thought there was a significant risk of it doing damage. Maybe in my ignorance, I didn’t. I assumed that as soon as anything problematic happened the withdrawal of medication would be reversed, as indeed has happened to some extent''.* | **Benefits of taking part (monitoring):** Described several times his perceived benefits of taking part- *''I’d be, not looked after, but I’d be checked up''. ''A year or two carrying on under monitored clinical conditions. I knew full well I wouldn’t be getting that back at home''.* | **Study outcome:** Describes being *''still on medication, which I will be on for a long time, I appreciate that. I’m on less than I was''*  **Polypharmacy:** *''I think there were 17 different pills I was on at one point. I’m still on the blood thinning ones, I can’t even remember what I’m on. The number, I was having to take them three times a day at one point. The fact that now in the morning I take four pills and that’s it.''*  **Understanding study results:** '*'the results, I think it’s been less successful in terms of reducing the pills than they maybe hoped for'',* | **Future research:** Describes research that would focus on the heart and [another existing health condition]: *''look at both and analyse how they interact?''* | **Perception of heart condition:** *''I still don’t really think I have a heart condition. I know my heart has been affected by my [pre-existing health condition] having a meltdown''*  **Relationship with study staff:** Refers to seeing the study coordinator and the benefit of being seen promptly. | **Compliance with medication:**  *''As soon as I came out, I just carried on doing the same routine I’d adopted in hospital'.”* |

**Positionality**

Our research team consisted of cardiologists and a nurse involved in the original TRED-HF study (BH, SKP, JC, RW), a patient representative (RM), trained nurses (KJ, JB), public health specialist (HW) and a qualitative researcher with experience in public involvement (VP). Although no member of the research team other than RM was living with dilated cardiomyopathy themselves, some were living with a diagnosed heart condition or had a family member who was. This helped to ‘ground’ our findings within lived experiences of, for instance, diagnosis, treatment, and management of a lifelong condition. Our varied backgrounds, personal and professional experiences will likely have influenced the study design and interpretation of findings, however, involving RM has helped us keep the research meaningful to any readers who are living with, or affected by, dilated cardiomyopathy.

**References**

1. NVivo qualitative data analysis software. QSR International Pty Ltd.; 2018.

2. Ritchie J, Lewis J, Nicholls CM, Ormston R. Qualitative research practice: A guide for social science students and researchers. SAGE; 2013.

3. Kvale S. The Qualitative Research Interview. *Journal of Phenomenological Psychology* 1983;**14**.
