# Supplementary material for: A qualitative exploration of participant and investigator perspectives from the TRED‐HF trial
Source: ESC Heart Fail. 2021 Aug 13;8(5):3760–8. doi: 10.1002/ehf2.13524 (PMC8497205; doi:10.1002/ehf2.13524)
Supplement: Supplementary file 3 — Data S3. Topic guide used with TRED‐HF participants and staff interviewed. [file EHF2-8-3760-s002.docx]

### Supplementary File 3: Interview topic guide

The topic guide was informed by available literature^1^ and included a number of prompts to help encourage interviewees to share their views. Prompts are indicated underneath each numbered question.

**Questions for TRED -HF participants:**

1. Tell me about yourself
   1. Heart condition/any other conditions?
   2. How did you hear about study?
2. Why did you decide to take part?
3. What did you expect from taking part?
   1. Any concerns?
   2. Did you think of any risks of taking part? (e.g. heart function)
   3. What would be an “acceptable” risk?
   4. What would “success” have looked like? (e.g. reducing dose, stopping all medications)
4. Tell me about your experience of taking part
   1. Who were your key contacts?
   2. What was the outcome for you?
5. At the start of the study, did you view yourself as still having a heart condition?
   1. What about at the end of the study?
6. At the start of the study, what did you think was the likelihood of you being able to stop taking your medication?
7. Before the start of the study, what impact did taking medications have on your quality of life?
   1. Did this change by the end of the study? How?
8. What do you understand about the results of the study?
9. Given your experience, if you had the time again would you take part? Why?
   1. If yes – so you have no regrets?
10. What should be the focus of future research on dilated cardiomyopathy

**Questions for TRED-HF investigators:**

1. Can you please tell me more about your role?
   1. Job title
   2. In the study
2. Have you worked on clinical trials before the TRED study?
   1. What was your role?
   2. When?
   3. Where?
   4. Was working on the TRED study any different?
3. Where did the idea of the TRED study come from?
4. Were any patients involved in designing the study?
5. What was being involved in the trial like for you?
   1. Were there any challenges?
   2. How about the experience of patients?
6. You mentioned earlier that your role in TRED was as [JOB TITLE]. Can you tell me more about what you did?
7. Have you discussed the results of the study with participants? Can you tell me more about this?
8. Are you aware that some participants have revealed that they are still no longer taking their medication? What do you think about that?
9. Is there anything you would do differently?
10. We’ve been asking participants what they think future research on dilated cardiomyopathy should focus on. What are your views?
11. Is there anything else you’d like to share with me?

**References**

1. Lavender V, Gibson F, Brownsdon A, Fern L, Whelan J, Pearce S. Health professional perceptions of communicating with adolescents and young adults about bone cancer clinical trial participation. *Supportive Care in Cancer* 2019;**27**:467–475.
