# Supplementary material for: A qualitative exploration of participant and investigator perspectives from the TRED‐HF trial
Source: ESC Heart Fail. 2021 Aug 13;8(5):3760–8. doi: 10.1002/ehf2.13524 (PMC8497205; doi:10.1002/ehf2.13524)
Supplement: Supplementary file 4 — Data S4. Diagrammatic representation of main themes and sub‐themes identified in framework analysis. [file EHF2-8-3760-s001.pptx]

## Slide 1
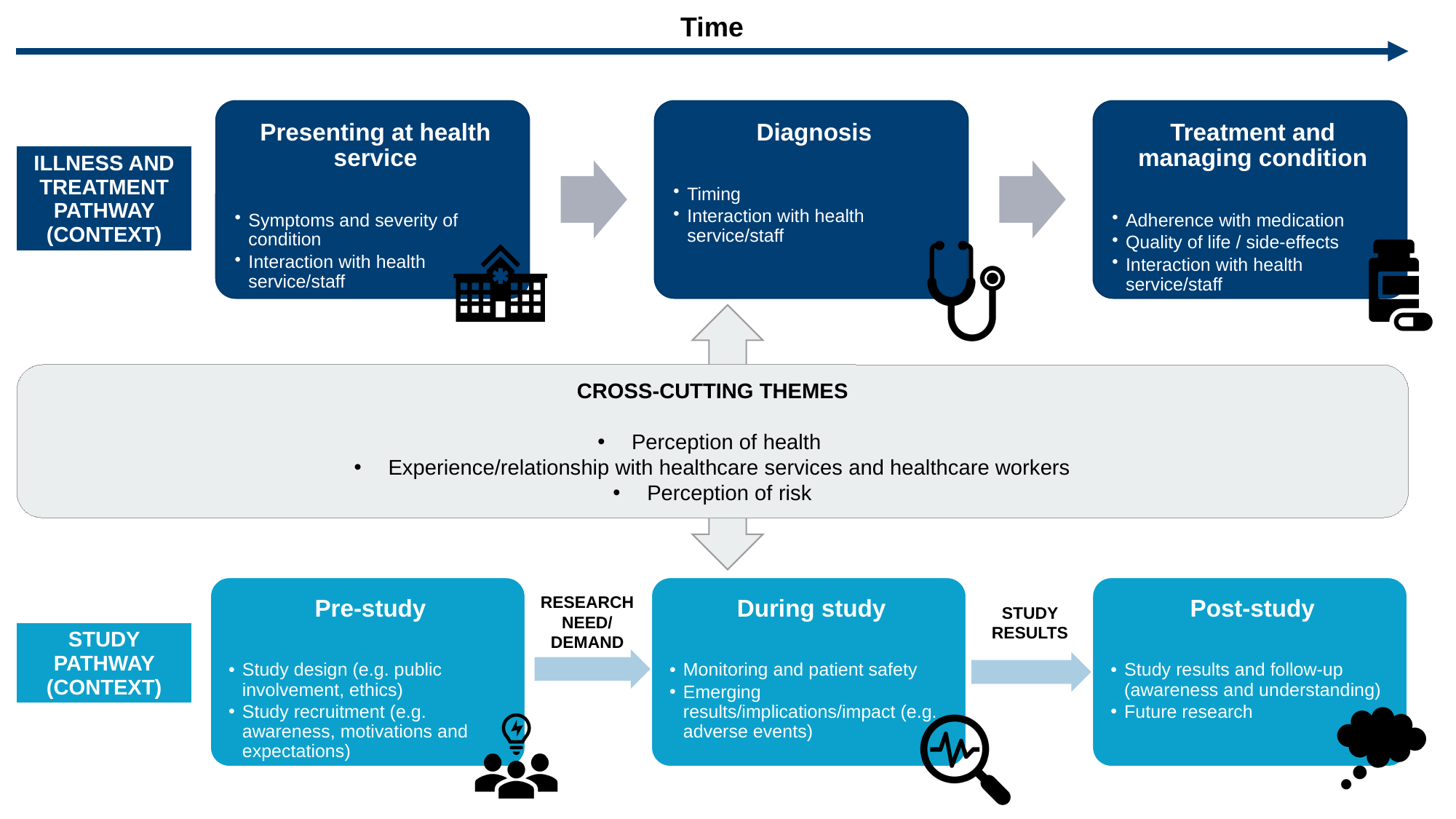

Time
| ILLNESS AND TREATMENT PATHWAY (CONTEXT) |
| --- |
CROSS-CUTTING THEMES
Perception of health
Experience/relationship with healthcare services and healthcare workers
Perception of risk
RESEARCH NEED/
DEMAND
STUDY RESULTS
| STUDY PATHWAY (CONTEXT) |
| --- |
